# Daily feeding frequency impacts muscle characteristics and fat deposition in finishing pigs associated with alterations in microbiota composition and bile acid profile

**DOI:** 10.3389/fmicb.2025.1510354

**Published:** 2025-01-28

**Authors:** Luga Hu, Huayu Tang, Zhaoxi Xie, Hongyu Yi, Lunjie Feng, Pan Zhou, Yong Zhang, Jingbo Liu, Xiang Ao, Jianchuan Zhou, Honglin Yan

**Affiliations:** ^1^School of Life Science and Engineering, Southwest University of Science and Technology, Mianyang, China; ^2^Feng Guangde Laboratory, Sichuan TQLS Group, Mianyang, Sichuan, China

**Keywords:** feeding frequency, myofiber type transformation, lipid deposition, microbiota, bile acids

## Abstract

**Introduction:**

Feeding frequency has been shown to affect growth and body composition of the host associated with gut microbiota. It remains unknown whether adjusting feeding frequency could effectively regulate both skeletal muscle development and whole-body lipid metabolism and thus affect carcass composition and feed conversion efficiency. Therefore, this study aimed to explore the effects of feeding frequency on muscle growth, fat deposition, cecal microbiota composition, and bile acid composition in finishing pigs.

**Methods:**

Sixteen Sichuan-Tibetan black pigs, with an initial weight of 121.50 ± 1.60 kg, were divided into two groups and fed either two meals (M2) or four meals (M4) per day. The trial lasted 30 days. The muscle fiber characteristics, lipid metabolism in adipose tissue, and cecal microbiota and bile acid composition were determined.

**Results:**

The present study revealed that pigs fed four meals exhibited a lower feed-to-gain ratio, abdominal fat weight, and average backfat thickness (*p* < 0.05), as well as a higher loin eye area (*p* = 0.09) and myofiber diameter in the *longissimus* muscle than their counterparts. The mRNA expression of slow-twitch fiber and myogenesis-associated genes in the *longissimus* muscle was upregulated, while lipid metabolism-related genes in the backfat were downregulated in the M4 group compared to the M2 group (*p* < 0.05). The M4 pigs exhibited higher abundances of Firmicutes, Actinobacteriota, *Bacillus*, *Clostridium_sensu_1*, and *Romboutsia*, and lower abundances of Spirochaetota, Verrucomicrobiota, Treponema, and Muribaculaceae in the cecal content than the M2 pigs (*p* < 0.05). A higher feeding frequency increased the levels of primary bile acids and decreased the concentrations of taurine-conjugated bile acids in the cecal content of pigs (*p* < 0.05).

**Conclusion:**

Our research suggested that the M4 feeding pattern, compared to the M2 pattern, promoted muscle growth and reduced fat deposition by enhancing fast- to slow-twitch fiber conversion and myogenesis in the muscle and repressing lipid metabolism in adipose tissue, associated with altered microbiota composition and bile acid profiles.

## 1 Introduction

As epidemiological studies (Wilkinson et al., 2020; Wei et al., 2018) have revealed the role of intermittent feeding/fasting in controlling body weight and composition in both humans and rodents, the effects of feeding frequency on growth rate and feed utilization efficiency of pigs have gained increasing research interest. The majority of the studies have revealed that adjusting feeding frequency could affect the growth rate of pigs, resulting in either improved or reduced feed efficiency (Gourley et al., 2020; Bruun et al., 2023; Ren et al., 2017). It has been shown that the regulation effects on the lipid deposition of feeding frequency contribute to the changes in feed efficiency (Zhang et al., 2022; Liu et al., 2017; Yan et al., 2020). It is well known that the skeletal muscle composes approximately 40 ~ 50% of body mass, and its development plays a key role in determining the growth rate and carcass composition of animals (Matarneh et al., 2021). Previous studies showed that the carcass composition of pigs was mainly influenced by the muscle-to-bone ratio when the body fat level was kept at a relatively constant range, implying that both skeletal muscle and fat deposition rates contribute to the feed utilization efficiency of animals (Ismail and Joo, 2017). However, only fragmentary information on the effects of feeding frequency on skeletal muscle growth showed that altered feeding frequency influenced muscle mass and protein expression related to glucose metabolism, energy production, and lipid utilization (Cao et al., 2022; Liu et al., 2017). Therefore, the effects and mechanisms of feeding frequency on skeletal muscle properties need to be further clarified.

The mammal’s gastrointestinal tract contains many commensal microbes providing beneficial metabolites that trigger host metabolic pathways. Hence, gut microbiota has been regarded as a ‘metabolic organ’ that can regulate host metabolism (Devin et al., 2016). The relationship between gut microbiota and skeletal muscle development and lipid metabolism of the host has been extensively studied in the past decade, showing that lipid metabolism and myofiber development are associated with gut microbiota (Wu et al., 2021). Our previous studies have demonstrated that the growth rate of the skeletal muscle and lipid metabolism of animals was decided by the gut microbiota and their metabolites, which was proved to mediate the effects of nutrition strategies that had on skeletal muscle development and body composition of pigs (Zhang et al., 2019). Accumulated evidence showed that the effects of feeding frequency on the feed conversion efficiency of pigs may be attributed to the regulation of gut microbiota composition and bile acid profile (Yan et al., 2016; Zhu et al., 2022). Previous studies have indicated that the bile acids acting as the signal molecules activate the farnesoid X receptor (FXR), resulting in the increased secretion of fibroblast growth factor (FGF) 15/19 from the gut into the blood (Kir et al., 2011; Ridaura et al., 2013). FGF15/19 has been shown to promote the skeletal muscle growth of the host (Devin et al., 2016). Hence, it can be hypothesized that feeding frequency may have a strong effect on regulating skeletal muscle development by shaping the gut microbiota composition and bile acid profile.

Owing to the effects that feeding frequency had on the growth and body composition of the host and the existing connection between gut microbiota and skeletal muscle properties and lipid deposition of the host, we hypothesized that adjusting feeding frequency could effectively regulate both skeletal muscle development and whole-body lipid metabolism and thus affect carcass composition and feed conversion efficiency of finishing pigs. These effects may be attributed to the changes in the composition of microbiota and bile acids. Sichuan–Tibetan black pigs, a newly developed obese pig breed from Sichuan province, China, are characterized by a fast growth rate but exhibit high-fat deposition during the late growth phase. Typically, during the finishing period, these pigs are fed two meals per day with a restricted feed allowance to control body composition and achieve relatively higher feed efficiency. However, it remains unknown whether higher or lower feeding frequency is more suitable for the Sichuan–Tibetan black pigs in the late growth phase. Therefore, the purpose of this study was to explore the effects of feeding frequency on growth performance, carcass traits, meat quality, myofiber characteristics, and protein deposition in skeletal muscle, lipid metabolism in adipose tissue, and bile acid profile and microbiota composition in the hindgut of Sichuan–Tibetan black pigs.

## 2 Materials and methods

The experimental protocols for the current study were approved by the Institutional Animal Care and Use Committee of Southwest University of Science and Technology (No. 20220106).

### 2.1 Animal housing and experimental design

Sixteen 210-day-old castrated Sichuan–Tibetan black pigs with an average initial body weight of 121.50 ± 1.60 kg were chosen for the present study and were randomly assigned to two treatment groups: the pigs were fed either with two meals (8:00 and 16:00) per day (M2 group) or with four meals (8:00, 12:00, 16:00, and 20:00) per day (M4 group). Each treatment had eight replicates; each replicate included one pig. The pigs were individually housed in a pen (1.5 m × 1.5 m) with a feeder and a nipple waterer in an environmentally controlled room. All the pigs were offered 2.60 kg of feed allowance per day. Pigs that were fed the M2 regimen received feed with one-half of their feed allowance per meal, and pigs that were fed the M4 regimen received a diet with one-fourth of the daily feed allowance per meal. Each meal for both the M2 and M4 groups lasted 2 h. At d0 and d30 of the trial, the pigs were weighed to record the initial and final weights and to calculate the average daily gain (ADG). The feed-to-gain ratio was calculated by dividing the fixed feed allowance (2.60 kg/d/pig) by ADG. The composition and nutrient levels of the used diet are shown in Supplementary Table S1. The feeding trial was conducted at the experimental unit of TQLS Group (Mianyang, Sichuan, China).

### 2.2 Sample collection

At d30 of the experiment, after 8 h of fasting, 10 mL of blood was collected from each pig in a non-anticoagulant tube, followed by centrifuging at 3,000 × *g* for 10 min at 4°C to obtain the serum samples, which were stored at −20°C for parameters measurements. Subsequently, all the pigs were slaughtered to collect samples of *longissimus* muscle, backfat, and cecal contents, which were snap-frozen in liquid nitrogen, then transferred to the laboratory, and stored at −80°C for further analyses.

### 2.3 Carcass traits and meat quality

After the pigs were slaughtered and exsanguinated, the hot carcass weight was recorded by weighing the remaining body without the head, hoof, tail, and viscera. The dressing percentage refers to the proportion of hot carcass weight in the live body weight of the pigs. The abdominal fat was completely collected from the belly and weighed to record abdominal fat weight. The average backfat thickness was the average value of backfat depth at the 1st *thoracic vertebra*, last thoracic vertebra, and last lumbar vertebra of the left side of the carcass.

The pH values of the longissimus muscle between the 6th and 7th ribs from the left side of the carcass were measured 45 min and 24 h post mortem (referred to as pH 45 min and pH 24 h) using a pH meter (PH-STAR, SFK-Technology, Denmark) according to the manufacturer’s instructions. The pork color including lightness (L*), redness (a*), and yellowness (b*) was measured as the mean of three readings of each muscle sample using a Minolta CR300 Chromameter (Minolta Camera, Osaka, Japan). Each chop of the *longissimus* muscle from pigs was weighed, vacuum-packed in a plastic bag, and cooked in a water bath at 75°C to reach an internal temperature of 70°C. The cooked samples were chilled down for 30 min, blotted dry, and weighed. The difference between pre-and post-cooking weights was divided by the precooked weight to calculate the cooking loss percentage. Two replicate samples were determined on each muscle sample.

### 2.4 Muscle morphology

A small piece (2 cm × 1 cm × 1 cm) of the *longissimus* muscle was collected and placed in a 4% paraformaldehyde solution for fixation. Upon the initiation of morphology determination, the muscle samples that were immersed in the solution were dehydrated and embedded in paraffin wax. Three slices with 5-μm thickness of each sample were cut and stained with hematoxylin and eosin to observe the muscle morphology. The stained slices were photographed using an Olympus CX41 microscope at 40× magnification. The Olympus Cell B software (Olympus) was used to determine myofiber characteristics. Muscle fibers from 10 random fields of each sample were chosen to measure and calculate the diameter and cross-sectional area of myofiber. The fiber number of each field was counted and converted to the myofiber density by dividing the area of the field. Myofiber density was expressed as the fiber number in 1 mm^2^ of muscle.

### 2.5 Reverse transcription quantitative PCR

Total RNA from the *longissimus* muscle and backfat was isolated using RNAiso Plus, and only high-quality RNA was further reverse-transcribed to synthesize cDNA using the PrimeScript RT kit (TakaRa, Chengdu, China) according to the manufacturer’s instructions. The RT-qPCR was performed on a CFX96 Touch Real-Time PCR Detection System (Bio-Rad Laboratories, Inc.) using the TB Green Premix Ex Taq II (TakaRa, Chengdu, China). Each reaction consisted of 5 μL TB Green Premix Ex Taq II, 0.5 μL forward primer (10 μM), 0.5 μL reverse primer (10 μM), 2 μL Milli-Q water, and 2 μL cDNA template in a total reaction system of 10 μL. The thermal cycling conditions were as follows: an initial denaturation and enzyme activation step at 95°C for 3 min, then 40 cycles of denaturation/annealing/extension and data acquisition (95°C for 30 s, 40 s at annealing temperature depending on primer), and melt curve analysis from 65 to 90°C with 0.5°C increment every 5 s. In the current study, PCR amplification efficiencies consistently ranged from 90 to 110% and were used to convert the Cq values into raw data. The ACTB was used as a reference gene. The primer sequences are listed in Supplementary Table S2.

### 2.6 Microbiota analysis of the cecal contents

Cecal contents from all the pigs were used to isolate bacterial genome DNA using a QiaAmp DNA Stool Mini Kit (Qiagen, Beijing, China) following the manufacturer’s instructions. The DNA quality was evaluated visually using agarose gel electrophoresis. The V4–V5 hypervariable region of the 16S rRNA gene was amplified by PCR using the forward primer (5’-GTGCCAGCMGCCGCGGTAA-3′) and the reverse primer (5’-CCGTCAATTCMTTTRAGTTT-3′) for each cecal content sample. All the Illumina libraries were constructed by purified pooled amplicon DNA with the Ovation Rapid DR Multiplex System 1–96 (NuGEN, San Carlos, CA) and were sequenced on the Illumina MiSeq platform using the PE250 sequencing strategy. After sequencing, the Illumina data were processed using the Mothur software to get the OTU abundance table and the OTU taxonomic assignment table, which were further processed using R Studio v3.4.1. The representative sequence of each OTU was annotated with its taxonomic information with the Silva (SSU123) 16S rRNA database^2^. The diversity of communities and bacterial abundances of taxa were analyzed using the Community Ecology Package vegan and phyloseq in R script. To minimize the biases caused by sequencing depth between samples, the number of sequences per sample was randomly subsampled to the minimum sequencing depth. The raw sequencing data can be accessed with the accession number PRJNA1073754 in the NCBI BioProject database.

### 2.7 Quantification of bile acids

The bile acids (BAs) in cecal contents were extracted according to the methods described previously (Fang et al., 2018). Briefly, 50 mg of cecal content samples was weighed and homogenized with 200 μL of a mixture of acetonitrile and methanol for 5 min. The first supernatant solution was transferred to a new vial after centrifugation at 20,000 × *g* for 20 min. The remaining pellets were then reconstituted with 200 μL of a mixture of acetonitrile and methanol for 5 min and centrifuged at 20,000 × *g* for 20 min to obtain the second supernatant solution, which was then transferred to the vial containing the first supernatant solution. Each combination of two supernatant solutions was further centrifuged at 20,000 × *g* for 10 min to obtain the test solution for UPLC/TQ-MS analysis. A Waters Xevo TQ-S LC/MS Mass Spectrometer (Waters, Milford, MA, United States) equipped with an ESI source was adopted to determine the BA profile in samples. The concentration of each BA in the samples was determined based on the series dilutions of standards, and good linearity was confirmed.

### 2.8 Statistical analysis

Pigs were considered the experimental unit for all analyses (*n* = 8 per treatment), and all data are presented as mean ± se. The parametric data including the data on growth performance, carcass traits, meat quality, myofiber characteristics, BA abundances, and gene expression were tested for significance using the Student’s *t*-test by SAS 9.4. For the bacterial data, alpha diversity and the relative abundances of taxa were compared using the Mann–Whitney *U*-test by SAS 9.4, and the intragroup statistic differences in beta diversity were assessed using permutational multivariate analysis of variance (PERMANOVA) with 999 permutations using R script. Spearman’s correlation between the top 30 genera and BAs and Pearson’s correlation between BAs and gene expression data were calculated using the ggcor R package. The Benjamini–Hochberg method was adopted to correct multiple comparisons. The adjusted *p*-values less than 0.05 were considered statistically significant.

## 3 Results

### 3.1 Growth performance

Pigs that were fed four meals per day exhibited a higher final weight (*p* = 0.06), and ADG was significantly higher than the twice-fed group (*p* < 0.05). Compared to the M2 group, pigs in the M4 group had a significantly decreased feed-to-gain ratio (*p* < 0.05) (Table 1).

**Table 1:** Effects of different feeding frequencies on the growth performance of Sichuan–Tibetan black pigs.

Items	M2	M4	*P*-value
Initial weight, (kg)	121.38 ± 1.40	121.63 ± 1.10	0.89
Final weight, (kg)	139.25 ± 1.34	143.00 ± 1.24	0.06
ADFI, (kg)	2.60 ± 0.01	2.60 ± 0.01	1.00
ADG, (kg)	0.60 ± 0.01	0.71 ± 0.02	<0.01
F/G	4.37 ± 0.07	3.67 ± 0.12	<0.01

The results are presented as mean ± se. *n* = 8.M2, pigs for two meals feeding per day; M4, pigs for four meals feeding per day. ADG, average daily gain; ADFI, average daily food intake; F/G, feed-to-gain ratio.

### 3.2 Carcass traits and meat quality

The hot carcass weight of pigs in the M4 group was significantly higher than that in the M2 group. Adjusting the feeding frequency of pigs from two meals to four meals per day significantly decreased abdominal fat weight and the average backfat thickness (*p* < 0.05). The higher feeding frequency tended to increase the loin eye area of Sichuan–Tibetan pigs (*p* = 0.09). There was a trend toward a higher value of lightness at 45 min post-mortem (*p* = 0.08) and cooking loss (*p* = 0.06) in the longissimus muscle in the M4 group compared to the M2 group (Table 2).

**Table 2:** Effects of feeding frequency on carcass traits and meat quality of Sichuan–Tibetan black pigs.

Item	M2	M4	*P*-value
Hot carcass weight (kg)	110.11 ± 1.33	114.11 ± 1.12	0.03
Dressing percentage (%)	79.01 ± 0.33	79.64 ± 0.10	0.11
Abdominal fat weight (kg)	3.61 ± 0.01	3.43 ± 0.01	<0.01
Average backfat thickness (mm)	48.96 ± 0.6	45.73 ± 0.84	0.01
Loin eye area (cm^2^)	42.45 ± 0.91	44.92 ± 1.00	0.09
*Longissimus* muscle			
L* _45 min_	40.87 ± 0.3	42.18 ± 0.65	0.08
a*_45 min_	1.01 ± 0.13	1.15 ± 0.15	0.51
b*_45 min_	6.92 ± 0.02	6.92 ± 0.23	1.00
L*_24 h_	48.16 ± 1.18	46.97 ± 0.62	0.39
a*_24 h_	1.36 ± 0.30	1.31 ± 0.26	0.90
b*_24 h_	8.31 ± 0.52	8.72 ± 0.51	0.58
pH_45 min_	5.69 ± 0.07	5.83 ± 0.07	0.18
pH_24 h_	5.58 ± 0.03	5.65 ± 0.10	0.16
Cooking loss (%)	39.5 ± 0.01	40.19 ± 0.31	0.06

The results are presented as mean ± se. *n* = 8. M2, pigs were given two meals per day; M4, pigs were given four meals per day. L*_45min_, a*_45min_, and b*_45min_ indicate the lightness, redness, and yellowness of the longissimus muscle at 45 min after slaughter; L*_24h_, a*_24h_, and b*_45min_ indicate the lightness, redness, and yellowness of the longissimus muscle at 24 h after slaughter.

### 3.3 Myofiber characteristics and myofiber type distribution in longissimus muscle

**Figure 1:**
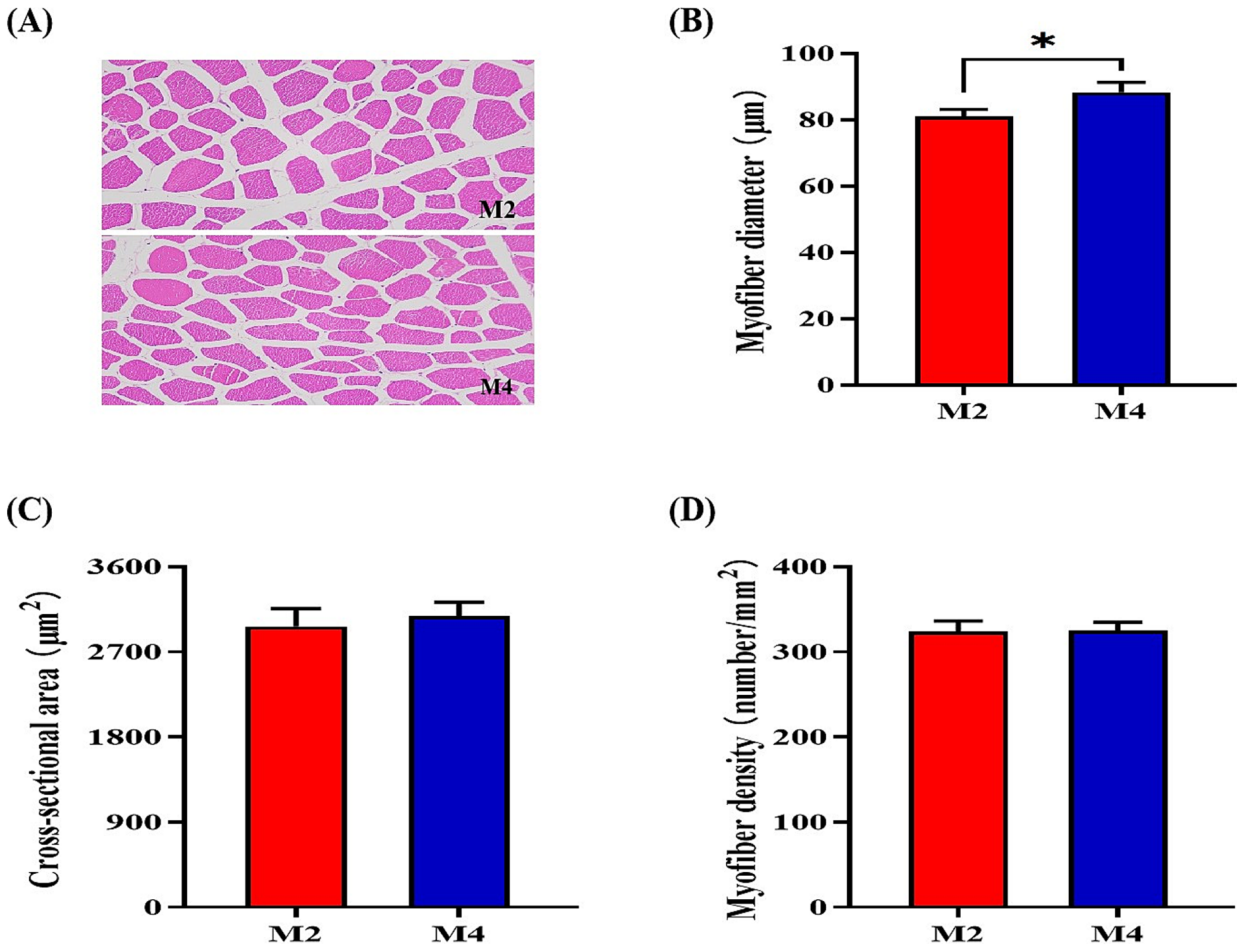
Effect of feeding frequency on myofiber characteristics in the longissimus muscle of Sichuan–Tibetan black pigs. **(A)** Representative images of myofiber staining; **(B)** myofiber diameter; **(C)** cross-sectional area; and **(D)** myofiber density. M2, pigs were fed two meals per day; M4, pigs were fed four meals per day. *p < 0.05, *n* = 8 for each group.

**Figure 2:**
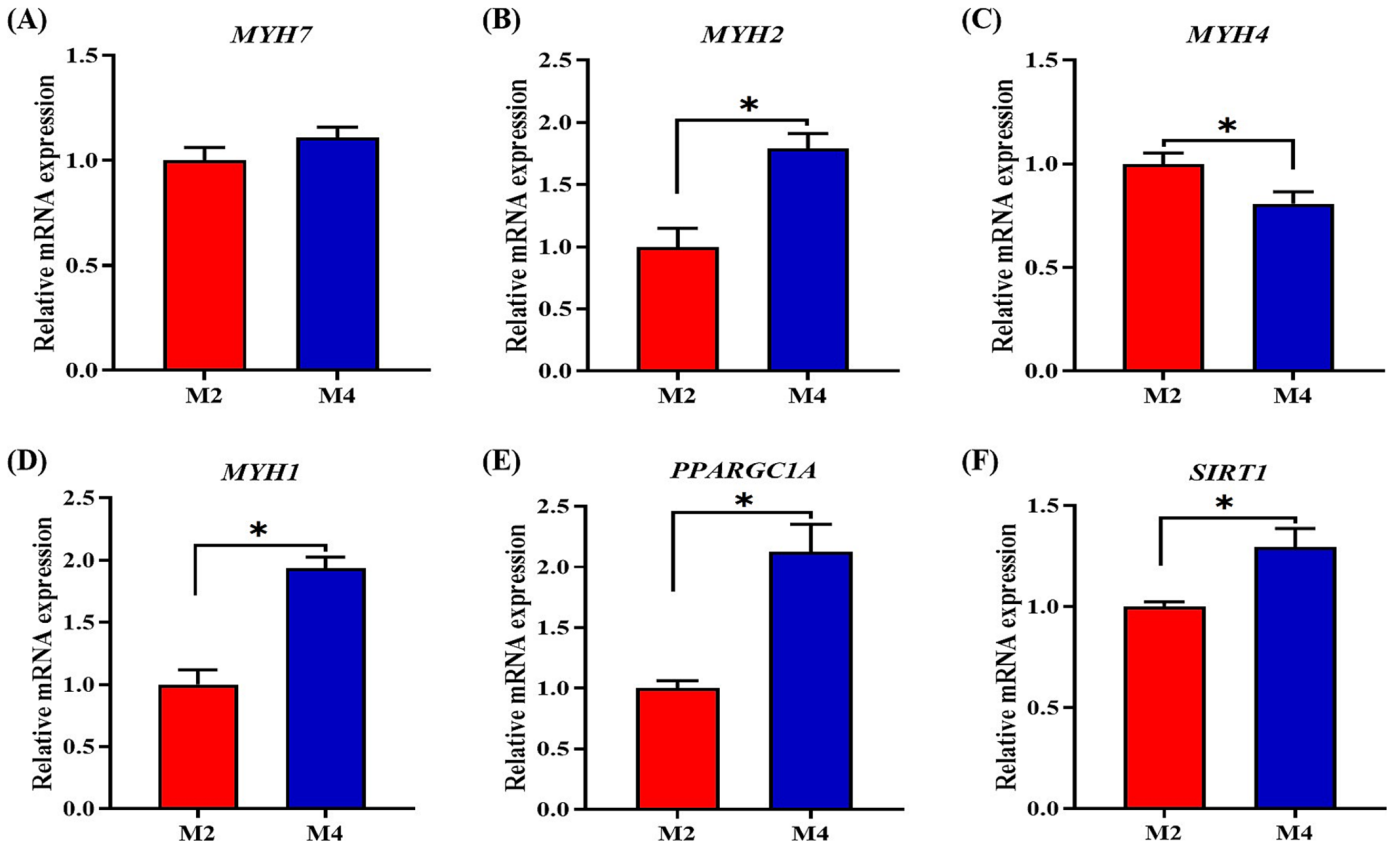
Effect of feeding frequency on the expression of genes related to myofiber type distribution and transformation in the *longissimus muscle* of Sichuan–Tibetan black pigs. **(A–D)** Myosin heavy chain isoforms, *MYH*, myosin heavy chain; **(E)** *PPARGC1A*, peroxisome proliferator-activated receptor gamma coactivator 1-alpha; and **(F)** *SIRT1*, silent information regulator 1. M2, pigs were fed two meals per day; M4, pigs were fed four meals per day. *p < 0.05, *n* = 8 for each group.

According to the observations of muscle morphology, there was no significant difference in cross-sectional area and density of muscle fibers (*p* > 0.05) between the M2 and M4 groups, but the higher feeding frequency significantly increased the myofiber diameter (*p* < 0.05) of pigs (Figure 1). Owing to the close relationship of muscle fiber type distribution and transformation with myofiber characteristics and meat quality, the mRNA expression of related genes in the longissimus muscle was determined. The expression of MYH2 and MYH1 was upregulated, and the abundance of MYH4 was downregulated in the longissimus muscle of pigs that were fed four meals compared to their counterparts that were fed two meals per day (*p* < 0.05, Figure 2). Increasing feeding frequency significantly elevated the mRNA abundances of PPARGC1A and SIRT1 (*p* < 0.05, Figure 2).

### 3.4 Myogenesis and protein metabolism in longissimus muscle

**Figure 3:**
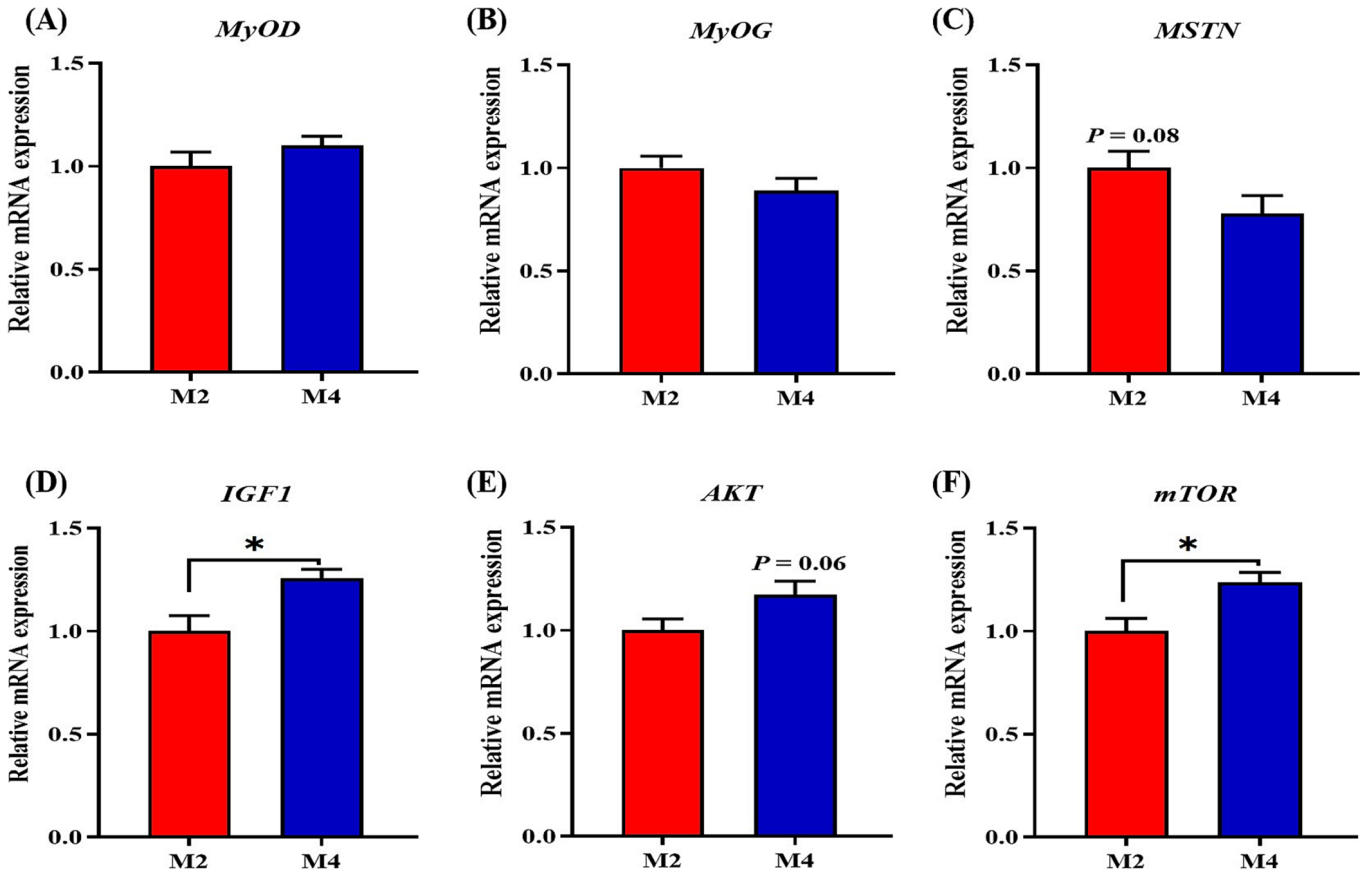
Effect of feeding frequency on the expression of genes related to myogenesis in *longissimus* muscle of Sichuan–Tibetan black pigs. **(A)** *MYOD*, myogenic differentiation 1; **(B)** *MYOG*, myogenin; **(C)** *MSTN*, myostatin; **(D)** *IGF1*, insulin-like growth factor 1; **(E)** *AKT*, protein kinase B; and **(F)** *mTOR*, mammalian target of rapamycin. M2, pigs were fed two meals per day; M4, pigs were fed four meals per day. *p < 0.05, *n* = 8 for each group.

To clarify whether the effects of feeding frequency on feed efficiency and carcass composition were associated with changes in muscle growth and protein deposition, the expression of genes related to myogenesis and protein deposition in longissimus muscle was measured. Compared to pigs in the M2 group, pigs in the M4 group exhibited higher mRNA abundances of IGF1 and mTOR in the longissimus muscle (*p* < 0.05, Figure 3). The higher feeding frequency tended to depress the mRNA expression of MSTN (*p* = 0.08, Figure 3).

### 3.5 Lipid metabolism-related gene expression in adipose tissue

**Figure 4:**
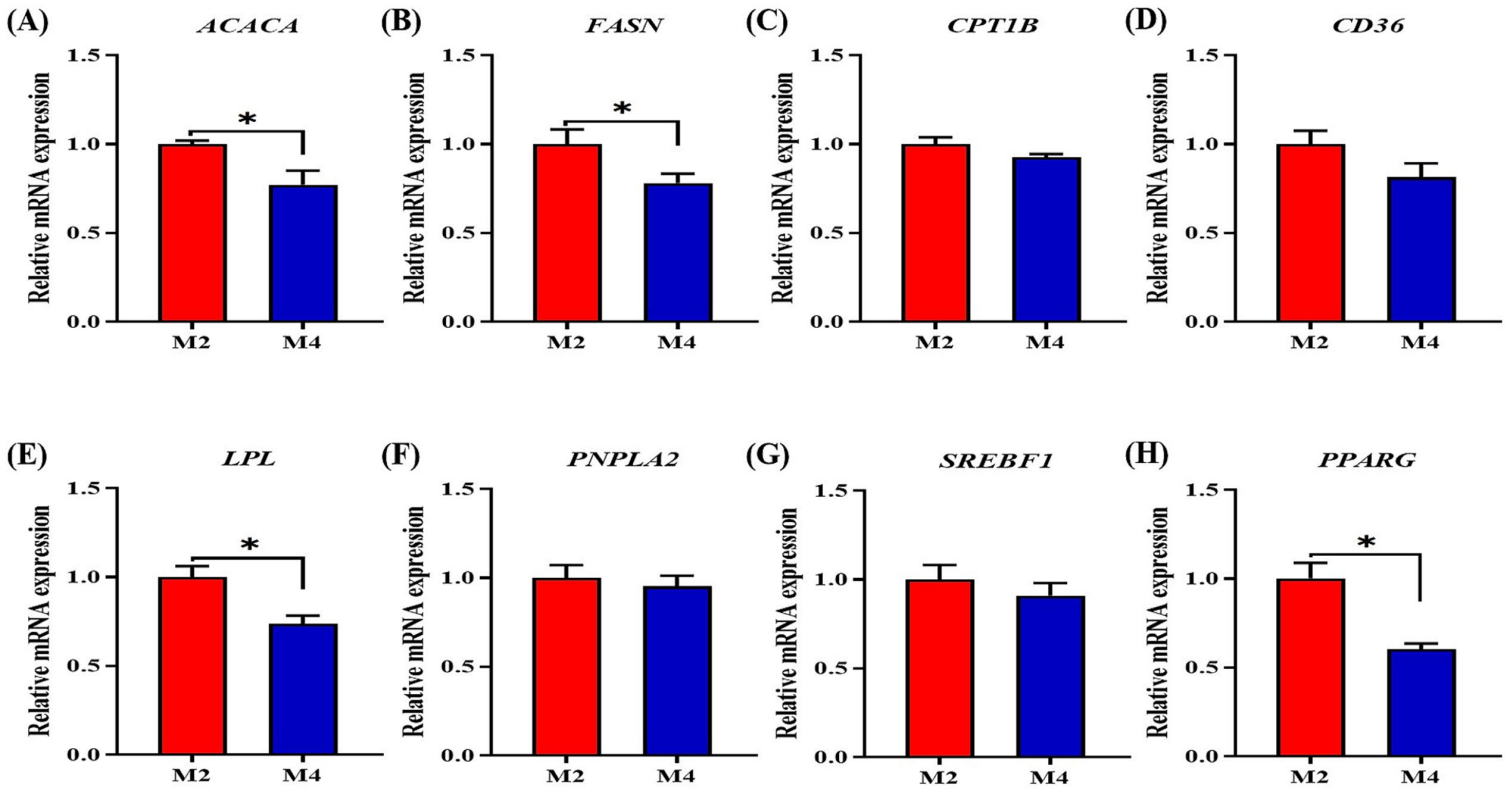
Effect of feeding frequency on lipid metabolism-related gene expression in the adipose tissue of Sichuan–Tibetan black pigs. **(A)** *ACACA*, acetyl-CoA carboxylase alpha; **(B)** *FASN*, fatty acid synthase; **(C)** *CPT1B*, carnitine palmitoyl transferase 1B; **(D)** *CD36*, CD36 molecule; **(E)** *LPL*, lipoprotein lipase; **(F)** *PNPLA2*, patatin-like phospholipase domain containing 2; **(G)** *SREBP1*, sterol regulatory element binding transcription factor 1; and **(H)** *PPARG*, peroxisome proliferator-activated receptor gamma. M2, pigs were fed two meals per day; M4, pigs were fed four meals per day. *p < 0.05, *n* = 8 for each group.

Increasing feeding frequency reduced fat mass in the back and belly of pigs in the present study; we thus further explored the alterations of gene expression related to lipid metabolism in the adipose tissue of pigs. The four-meal feeding regimen significantly decreased the mRNA expression of ACACA and FASN in backfat tissue compared to the two-meal feeding regimen (*p* < 0.05, Figure 4). Increasing feeding frequency significantly downregulated the mRNA expression of LPL, SREBP1, and PPARG in the backfat tissue of pigs compared to those in the M2 group (*p* < 0.05, Figure 4).

### 3.6 Microbiota composition and BA profile in the cecum

**Figure 5:**
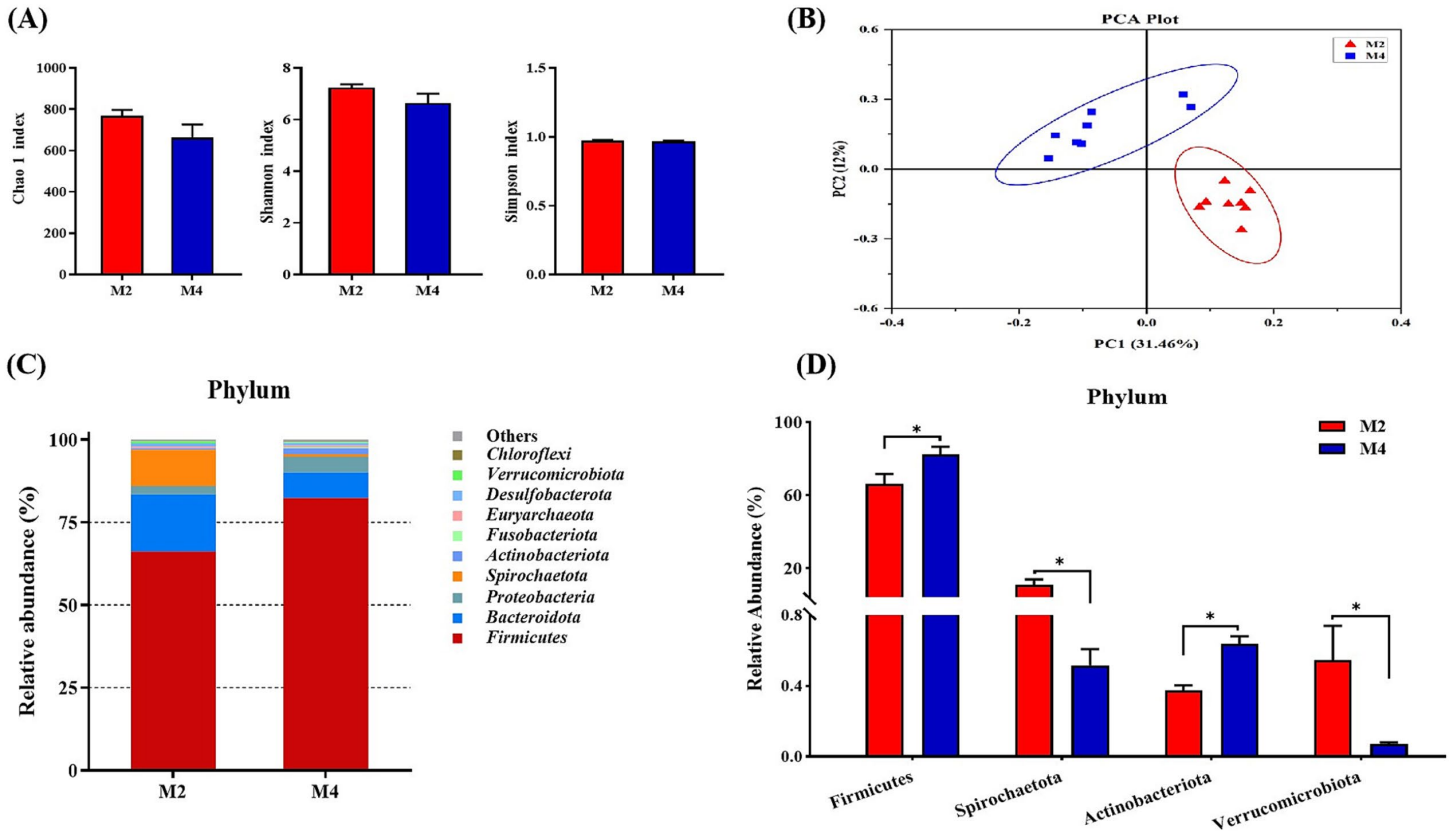
Effect of feeding frequency on cecal microbiota composition of Sichuan–Tibetan black pigs. **(A)** Alpha-diversity indices; **(B)** principal component analysis (PCA) plot based on OTU composition; **(C)** distribution of top 10 phyla in both groups; and **(D)** relative abundances of differentiated phyla between groups. M2, pigs were fed two meals per day; M4, pigs were fed four meals per day. *p < 0.05, *n* = 8 for each group.

**Figure 6:**
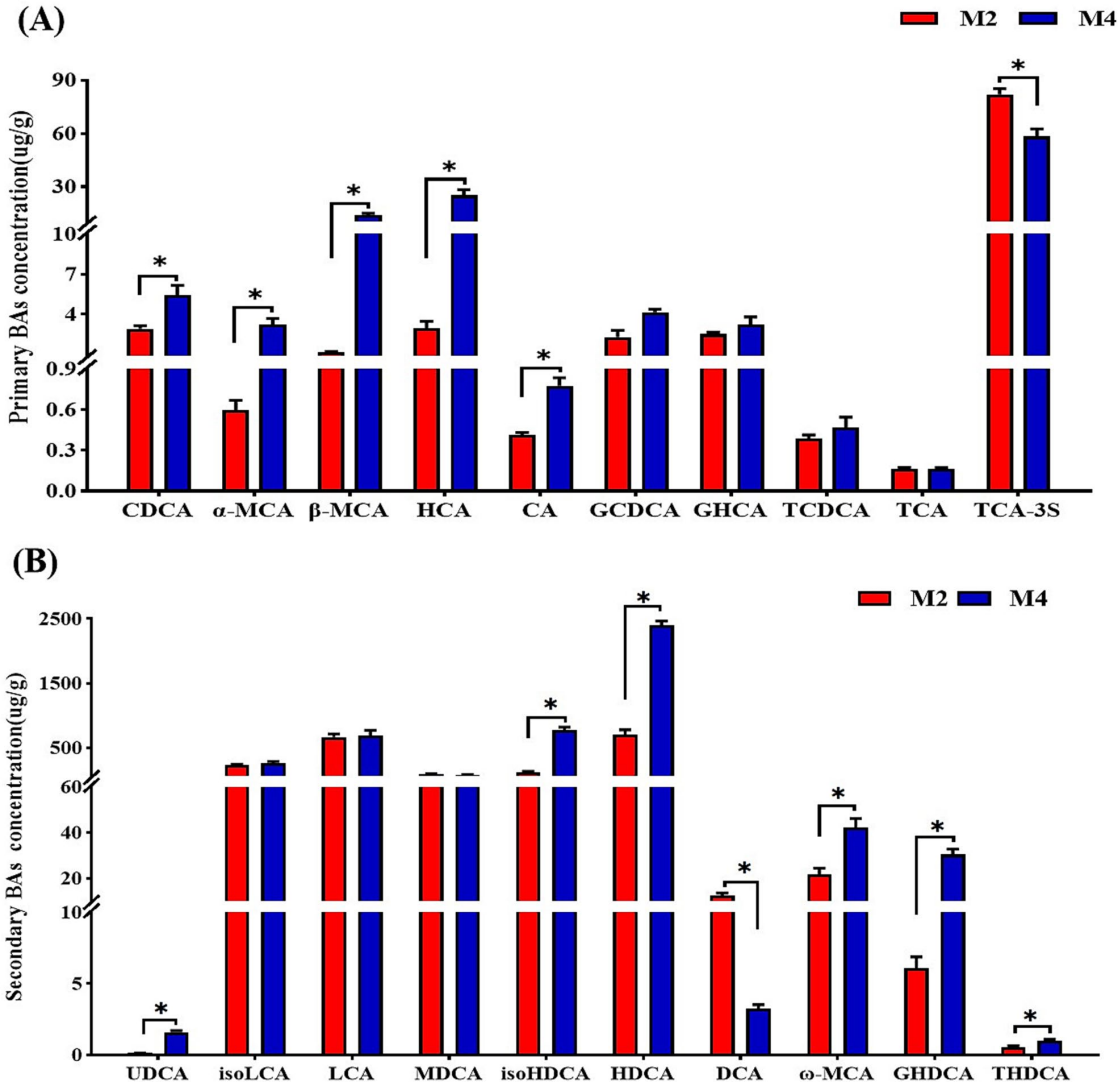
Effect of feeding frequency on individual primary bile acids **(A)** and secondary bile acids **(B)** concentrations in the cecal content of Sichuan–Tibetan black pigs. CDCA, chenodeoxycholic acid; α-MCA, α-muricholic acid; *β*-MCA, β-muricholic acid; HCA, hyocholic acid CA, cholic acid; GCDCA, glycochenodeoxycholic acid; GHCA, glycohyocholic acid; TCDCA, taurochenodeoxycholic acid; TCA, taurocholic acid; TCA, taurocholic acid-3-sulfate; UDCA, ursodeoxycholic acid; isoLCA, isolithocholic acid; LCA, lithocholic acid; MDCA, murideoxycholic acid; isoHDCA, isohyodeoxycholic acid; HDCA, hyodeoxycholic acid; βDCA, epideoxycholic acid; DCA, deoxycholic acid; *ω*-MCA, ω-muricholic acid; GHDCA, glycohyodeoxycholic acid; THDCA, taurohyodeoxycholic acid. M2, pigs were fed two meals per day; M4, pigs were fed four meals per day. *p < 0.05, *n* = 8 for each group.

The microbiota composition and BA profile in the cecal contents were determined to verify the critical role of microbiota in the effects of feeding frequency on the host. The results related to alpha diversity, beta diversity, and taxonomic distribution are presented in Figures 5, 6. There were no significant differences in alpha-diversity indices between the two groups (Figure 5A). A principal coordinate analysis (PCoA) based on the Bray–Curtis distance metric showed that the samples in the M2 group were clearly separated from the M4 group, indicating that altering feeding frequency could shift the gut microbiota composition of Sichuan–Tibetan pigs (Figure 5B). The microbiota composition at the phylum level was analyzed, and the results are listed in Figures 5C,D. The most dominant phylum was *Firmicutes* in all the samples (Figure 5C and Supplementary Table S3). Pigs that were fed four meals per day exhibited higher abundances of *Firmicutes* and *Actinobacteriota*, and lower abundances of *Spirochaetota* and *Verrucomicrobiota* than those fed two meals per day (*p* < 0.05, Figure 5D). At the genus level, the most predominant taxa in the M2 and M4 groups were *Treponema* and *Streptococcus*, respectively (Supplementary Figure S1A and Supplementary Table S4). The relative abundance of *Bacillus*, *Clostridium_sensu_1*, and *Romboutsia* was significantly higher in the cecum digesta of the M4 group than the M2 group. The higher feeding frequency significantly decreased the abundance of *Treponema* and *Muribaculaceae* in the cecum content of pigs (*p* < 0.05, Supplementary Figure S1B).

The BA profile in cecal content was further determined with LC–MS-based targeted metabolomics. It was found that increased feeding frequency significantly improved the concentrations of primary BAs including CDCA, *α*-MCA, *β*-MCA, HCA, and CA compared to the M2 group (*p* < 0.05, Figure 6A). The higher feeding frequency significantly increased the levels of secondary bile acids including UDCA, isoHDCA, HDCA, *ω*-MCA, GHDCA, and THDCA, and decreased the concentration of DCA in the cecal content of pigs (*p* < 0.05, Figure 6B). Analyzing the content of each type of bile acid revealed that the higher feeding frequency significantly increased the content of primary bile acids and decreased the content of taurine-conjugated bile acids (*p* < 0.05, Supplementary Figure S2), and there was a tendency toward a higher content of glycine-conjugated bile acids in the cecal content of pigs in the M4 group compared to those in the M2 group (*p* = 0.08, Supplementary Figure S2).

### 3.7 Correlation of intestinal microbiota with BAs and association between BAs and gene expression

**Figure 7:**
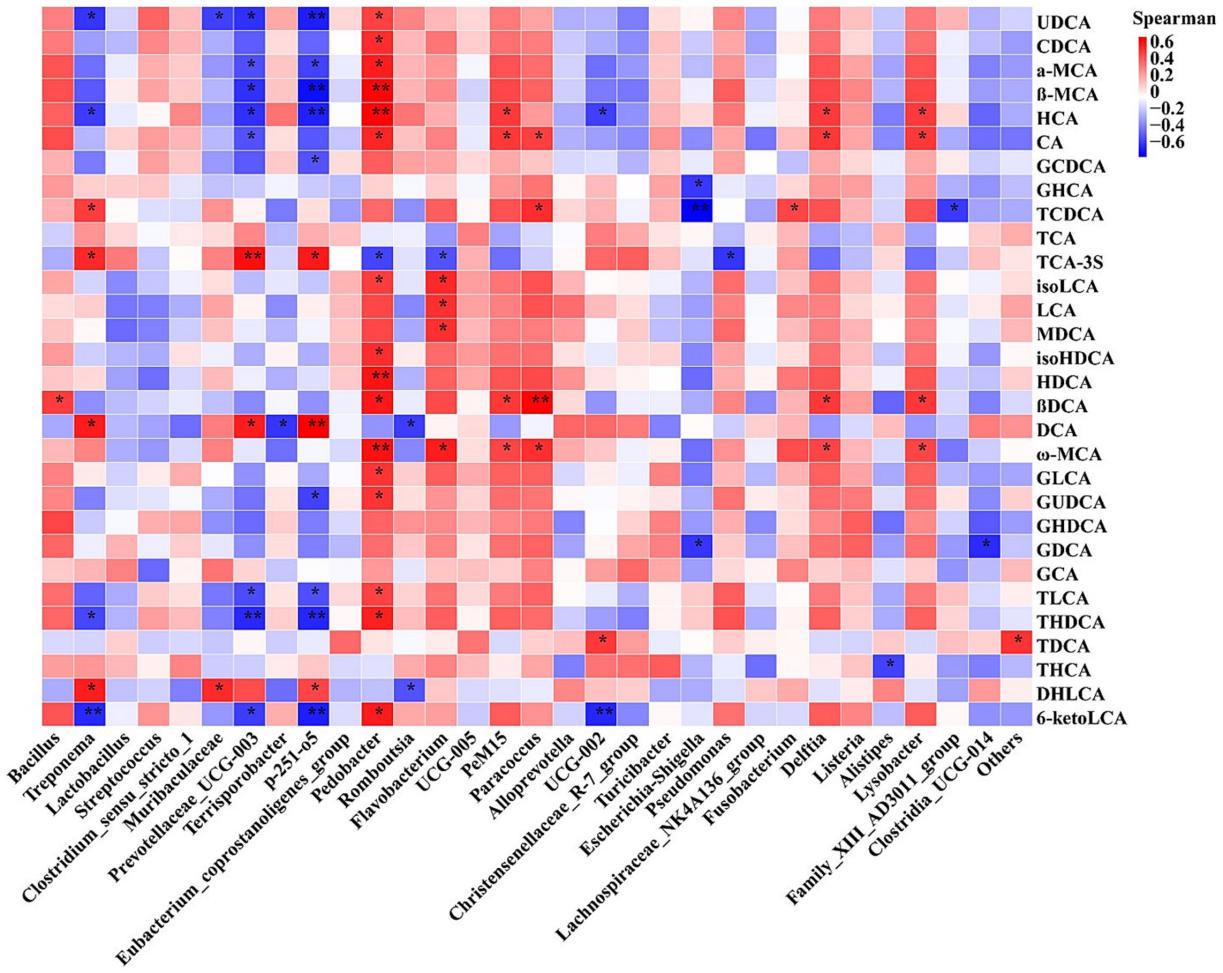
Heatmap of Spearman’s correlation between relative abundances of the top 30 genera and concentration of top 30 bile acids. CDCA, chenodeoxycholic acid; α-MCA, α-muricholic acid; β-MCA, β-muricholic acid; HCA, hyocholic acidCA, cholic acid; GCDCA, glycochenodeoxycholic acid; GHCA, glycohyocholic acid; TCDCA, taurochenodeoxycholic acid; TCA, taurocholic acid; TCA, taurocholic acid-3-sulfate; UDCA, ursodeoxycholic acid; isoLCA, isolithocholic acid; LCA, lithocholic acid; MDCA, murideoxycholic acid; isoHDCA, isohyodeoxycholic acid; HDCA, hyodeoxycholic acid; βDCA, epideoxycholic acid; DCA, deoxycholic acid; ω-MCA, ω-muricholic acid; GHDCA, glycohyodeoxycholic acid; THDCA, taurohyodeoxycholic acid; GLCA, glycolithocholic acid; GUDCA, glycoursodeoxycholic acid; GDCA, glycodeoxycholic acid; GCA, glycocholic acid; TLCA, taurolithocholic acid; TDCA, taurodeoxycholic acid; THCA, taurohyocholic acid; DHLCA, dehydrolithocholic acid; 6-ketoLCA, 6-ketolithocholic acid. M2, pigs were fed two meals per day; M4, pigs were fed four meals per day.*p < 0.05, **p < 0.01.

The above-mentioned results showed that feeding frequency altered the expression of genes related to myogenesis in muscle and lipid metabolism in adipose tissue, intestinal microbiota composition, and BA profile; thus, the correlation of intestinal microbiota with BAs and the association between BAs and gene expression were analyzed. Spearman’s correlation analysis (Figure 7) revealed that the relative abundance of *Pedobacter* was significantly positively correlated with the levels of UDCA, CDCA, *α*-MCA, *β*-MCA, HCA, CA, isoHDCA, HDCA, β-DCA, ω-MCA, GLCA, GUDCA, TLCA, THDCA, and 6-ketoLCA; the relative abundances of *PeM15*, *Paracoccus*, *Delftia*, and *Lysobacter* were significantly positively correlated with the levels of CA, β-DCA, and ω-MCA; the relative abundance of *Bacillus* was significantly positively correlated with the levels of β-DCA; the relative abundance of *Treponema* was significantly positively correlated with TCDCA, TCA-3S, DCA, and DHLCA and negatively correlated with UDCA, HCA, and 6-ketoLCA; the relative abundances of *p-251-o5* and *Prevotellaceae*_UCG-003 were significantly positively correlated with the levels of TCA-3S and DCA and negatively correlated with the concentrations of UDCA, α-MCA, β-MCA, HCA, TLCA, THDCA, and 6-ketoLCA; and the relative abundance of *Escherichia–Shigella* was negatively correlated with levels of GHCA, TCDC, and GDCA (Figure 7). The Pearson correlation analysis (Supplementary Figure S3) revealed that there were significant correlations between the abundances of individual BAs and the mRNA expression of genes related to myofiber transformation and myogenesis and genes related to lipid metabolism. The detailed results are described in Supplementary results section.

## 4 Discussion

Since the outbreak of African swine fever (ASF) in China in 2018, the stock of commercial pigs and sows dropped quickly, resulting in an increase in heavyweight pigs because the number of pigs required to produce a given quantity of pork is reduced and the fixed production cost is diluted (Guan et al., 2023). Sichuan–Tibetan black pigs are commonly raised to a high marketing weight of approximately 140 kg to adapt to the situation of the ASF pandemic and to pursue the production of high-quality pork. However, similar to the commercial pig breed, a higher marketing weight reduced the feed efficiency and lean deposition as well as accelerated fat deposition of Sichuan–Tibetan black pigs in the late finishing phase (Wu et al., 2017). To combat the drawback of the increased marketing weight of pigs, measures such as restricting feed intake and altering feeding frequency have been adopted in the practical production of Sichuan–Tibetan pigs. However, there is still a lack of experience and scientific knowledge regarding the setting of feeding frequency in fattening Sichuan–Tibetan pigs.

Changing feeding frequency has been shown to affect the partitioning of energy to lean and fat tissue deposition and thus regulate the feed conversion efficiency of meat-producing animals (Cao et al., 2022; Chaix et al., 2014). However, it is still inconclusive whether either higher or lower feeding frequency could improve feed conversion efficiency. Previous studies had inconsistent results about the effects of the higher feeding frequency on pig growth performance. Both studies from other groups and our previous study found that the duration of each meal determined the outcome of feeding frequency on feed conversion efficiency, showing improved feed conversion rate in pigs that were fed for two 60-min meals (Liu et al., 2017; Yan et al., 2021). In addition, it was found that the effects of feeding frequency on the growth performance of pigs, in particular on feed conversion rate, may largely depend on the feed allowance. Previous studies offering the same feed allowance to growing-finishing pigs fed at a higher or lower feeding frequency have demonstrated that the higher feeding frequency could improve growth performance including ADG and feed conversion efficiency of pigs (Jia et al., 2021; Schneider et al., 2011). Consistent with the findings in finishing pigs with fixed feed allowance, in the present study, Sichuan–Tibetan pigs that were fed four meals per day exhibited higher final weight, ADG, and feed conversion rate than the pigs that were fed two meals per day. However, low feed efficiency was found in the high-fat diet-fed pigs fed at a higher feeding frequency under the condition of fixed feed allowance in our previous study. The contradictory results may be attributed to the differences in the genetic background of pigs, the limit-feeding extent, the duration of each meal, and the setting of feeding frequencies between studies. Therefore, these factors should be considered in drawing conclusions about the effect that feeding frequency had on the growth performance of pigs, and the interactions between feeding frequency and these factors could be clarified in future studies.

Energy partition patterns determined the feed conversion efficiency of meat-producing animals (Moloney et al., 2008). In the late phase of growing, the energy partitioning between lean and fat mass is critical for the feed conversion rate of pigs. Even though the effects of feeding frequency on feed efficiency of pigs were inconsistent, altered feeding frequency improved feed efficiency and was associated with reduced carcass fat deposition (Colpoys et al., 2016; Yan et al., 2021). Consistent with this finding, our study found that the higher feeding frequency reduced abdominal fat weight and the average backfat thickness of Sichuan–Tibetan pigs. Moreover, in this study, the higher feeding frequency tended to increase the loin eye area and significantly increased the hot carcass weight, implying the enhanced lean mass and muscle growth rate in pigs that were fed the higher feeding frequency. This is consistent with previous findings in suckling pigs, where higher feeding frequency increased lean deposition and promoted muscle development (Liu et al., 2017).

Both the size of the individual fibers and/or the total number of fibers are known to be important determinants of muscle mass (Henckel et al., 1997). Previous studies showed that the number of muscle fibers was positively correlated with carcass weight and loin eye area of pigs, and pig littermates with a high fiber number or density tended to grow faster and more efficiently than those with a low fiber number or density in the skeletal muscle (Dwyer et al., 1993; Berry et al., 2018). Consistently, in the present study, the myofiber diameter of the longissimus muscle was higher in the M4 pigs, which had a higher growth rate and loin eye area. The composition of skeletal muscle fiber types in pigs, including four different myosin heavy chain (MyHC) isoforms (I, IIa, IIx, and IIb), was related to muscle characteristics and pork quality (Lefaucheur et al., 2002). Normally, MyHC differentiation was accompanied by myofiber density; the fiber density was highly correlated to the distribution of muscle fiber type (Rahman, 2007). In the present study, the mRNA expression of MYH2 and MYH1 was increased, and the mRNA expression of MYH4 was decreased in the longissimus muscle of the M4 pigs compared to the M2 pigs, confirming the findings of a previous study that the percentage of type IIa fiber was positively correlated with the muscle fiber density and loin eye area.

Both SIRT1 and PPARGC1A have been identified to promote the transformation of myofiber from fast-twitch to slow-twitch types (Wen et al., 2020). In the present study, the higher feeding frequency increased the mRNA expression of PPARGC1A and SIRT1 in the longissimus muscle of Sichuan–Tibetan pigs, implying that the PGC1α-induced myofiber type conversion may be a driving force for the accelerated myofiber differentiation and muscle mass growth (Huh et al., 2014). There are several regulators that positively or negatively affect the muscle growth of animals. The IGF1-myostatin system, the downstream molecules of PGC1α, has been demonstrated to play a critical role in diet-or exercise-induced muscle growth (Schiaffino et al., 2013). IGF1 is a positive regulator, and myostatin is a negative regulator of muscle growth. In this study, the higher feeding frequency significantly increased the expression of *IGF1* and *mTOR* and tended to decrease the mRNA expression of MSTN in the longissimus muscle, implying that the regulation of the IGF1-myostatin system by PGC1α could be mediating the effect of increased feeding frequency that primarily acts on enhancing muscle mass. The lipid deposition in carcass was mainly driven by several factors that are responsible for *de novo* fatty acid synthesis (ACACA, FASN, and SREBP1), fatty acid uptake (LPL and CD36), lipogenesis (PPARG), and lipolytic (CPT1B and PNPLA2). In the present study, a four-meal feeding regimen significantly decreased the mRNA expression of ACACA, FASN, SREBP1, and PPARGC1A in adipose tissue compared to the two-meal feeding regimen. This is consistent with the previous findings that the fatty acid metabolism pathways were suppressed with an increase in feeding frequency (Zhang et al., 2022; Yan et al., 2020). Moreover, increased lipogenesis was observed in the animals fed fewer meals daily, as more circulating nutrients were not efficiently in muscle tissue, leaving more nutrients available for adipose tissue or the liver for fat synthesis (Chasse et al., 2021). The higher circulation levels of glucose, non-esterified fatty acids, and very low-density lipoprotein cholesterol were also found in the higher feeding frequency-fed pigs by the previous study (Zhang et al., 2022). Our results strongly support the regulatory roles of feeding frequency on muscle growth and lipid metabolism, which greatly contributes to the regulation of carcass traits and feed efficiency through daily eating patterns in pigs.

The important role of gut microbiota in regulating muscle growth and fat deposition in pigs has been revealed by previous studies using germ-free animal models (Yan et al., 2016; Wen et al., 2019; Qi et al., 2021), showing that the absence of gut microbiota significantly affects the proportions of slow-twitch and fast-twitch fibers in the *longissimus* muscle of pigs, and the transplantation of gut microbiota from obese pigs could transfer the phenotypes related to myofiber characteristics and lipid metabolism from pig donors to mice recipients (Yan et al., 2016). In the present study, the gut microbiota of pigs in the M4 group was clearly separated from those in the M2 group, and the abundances of phyla Spirochaetes and Verrucomicrobiota were significantly lower in cecal contents of the M4 pigs than their counterparts, which is consistent with the previous finding that the gut microbiome of lean pigs harbored lower abundance of Spirochaetes than that of obese pigs (Yan et al., 2016). The abundance of Spirochaetes has been negatively correlated with the muscle mass of pigs as the decrease in fecal Spirochaetes level was found in myostatin gene-mutated pigs that exhibited muscle hypertrophy (Pei et al., 2021). The gut microbiota–muscle axis was further confirmed by a previous study using a myostatin deletion pig model and fecal microbiota transplantation, which demonstrated that MSTN deficiency stimulated skeletal muscle growth and promoted the growth of genera Romboutsia and Clostridium_sensu_stricto_1. These genera produce microbial-derived organic acids and derivatives, such as short-chain fatty acids and bile acids (Pei et al., 2021; Luo et al., 2023). Consistently, in this study, the M4 pigs with accelerated muscle growth and feed efficiency exhibited higher abundances of genera Romboutsia and Clostridium_sensu_stricto_1 in cecal contents. In addition, four meal-fed pigs, which had a higher loin eye area and lower adiposity, were found to have a lower level of the genus *Treponema* in cecal content than M2 pigs in the current study, which was inconsistent with the previous study, showing that the taxa annotated to the *Treponema* were negatively associated with fatness traits of pigs. Myostatin deletion has been shown to decrease the abundance of *Treponema* in jejunal content but increase the abundance of *Treponema* in the feces of pigs (Luo et al., 2023; Pei et al., 2021), implying that the contradictory results may be attributed to the difference in sample sites for microbiota analysis.

Accumulating evidence suggests that gut microbiota regulated muscle development and fat deposition through metabolite–host interactions (Qiu et al., 2021; Lahiri et al., 2019; Chen et al., 2021). A previous study showed that the changed gut microbiome and coupled altered stool BA profile mediated the protective effects of restricted time feeding on diet-induced obesity in mice (Chaix and Zarrinpar, 2015). Therefore, we hypothesized that the perturbed microbiota-elicited changes in the BA profile would be responsible for the effects of the four-meal feeding pattern on the growth and carcass phenotypes of pigs. In this study, four-meal feeding significantly increased the total amounts of primary bile acids and decreased the total amounts of taurocholic bile acids, suggesting the enhanced bacterial bile salt hydrolase activity in the cecum of the M4 pigs (De Smet et al., 1998). Moreover, in the present study, the concentrations of primary bile acids CDCA, α-MCA, β-MCA, HCA, and CA, as well as the concentrations of secondary bile acids UDCA, LCA, HDCA, GHDCA, THDCA, isoHDCA, and ω-MCA, were significantly higher in the M4 group than those in the M2 group, which is consistent with the previous finding that intermittent fasting decreased obesity by enhancing the BA metabolism with higher primary and secondary BA levels in the gut lumen and serum. It has been shown that the microbe-derived metabolites are responsible for the diet-or feeding pattern-induced changes in growth and metabolism phenotypes (Janeckova et al., 2019; Chang et al., 2018). Altered BA profiles have been associated with the improvement effects of feeding frequency on the feed efficiency of pigs (Yan et al., 2021). Therefore, we further analyzed the correlation between bile acids and the mRNA expression of genes related to myofiber transformation and lipid metabolism. The results showed the positive correlations between individual bile acids and the expression of *MYH7*, *MYH2*, *MYH1*, *PPARGC1A,* and *SIRT1*, as well as the negative correlations between individual bile acids and the expression of fatty acid metabolism-related genes. Previous studies have shown that BAs are signaling molecules that regulate host skeletal muscle synthesis and lipid metabolism by binding to their receptors, such as membrane G-protein-coupled receptor 5 (TGR5) and the nuclear farnesoid X receptor (FXR) (Sun et al., 2018; Sasaki et al., 2018). BAs/FXR-induced FGF19 secretion into circulation has been shown to promote skeletal muscle mass accretion and regulate its function (Qiu et al., 2021). However, it remains unknown whether gut microbiota-BA-FXR-FGF19 signaling mediates the effects of feeding frequency on myofiber type conversion and lipid metabolism in pigs, which should be addressed in further studies.

## 5 Conclusion

In the present study, for the Sichuan–Tibetan black pigs, the four-meal feeding pattern improved growth rate, feed efficiency, and carcass lean mass by promoting the expression of genes related to myofiber transformation and differentiation in skeletal muscle and inhibiting the expression of fatty acid synthesis-related genes in adipose tissue, which may be associated with the changes in microbiota composition and bile acid profile.

## Data Availability

The original contributions presented in the study are publicly available. This data can be found at: https://www.ncbi.nlm.nih.gov/bioproject, accession number PRJNA1073754. Further inquiries can be directed to the corresponding author.

## Footnotes

^2^ http://www.arb-silva.de/
