# Supplementary material for: Daily feeding frequency impacts muscle characteristics and fat deposition in finishing pigs associated with alterations in microbiota composition and bile acid profile
Source: Front Microbiol. 2025 Jan 28;16:1510354. doi: 10.3389/fmicb.2025.1510354 (PMC11813218; doi:10.3389/fmicb.2025.1510354)
Supplement: Supplementary file 1 [file Table_1.DOCX]

Supplementary material

**Supplementary results**

The concentrations of UDCA, CDCA, α-MCA, β-MCA, HCA, CA, GCDCA, TLCA, THDCA, and 6-ketoLCA were significantly positively correlated with the expression of the myofiber transformation-related genes *MYH2*, *MYH1* ,and *PPARGC1A*；the levels of UDCA, CDCA, α-MCA, HCA, CA, GCDCA, GLCA, and TLCA were significantly positively correlated with the expression of *MYH7*; the concentrations of UDCA, α-MCA, β-MCA, HCA, and 6-ketoLCA were significantly positively correlated with the expression of *SIRT1* and *IGF1*; the levels of TCA-3S, DCA, and THCA were significantly positively correlated with the expression of *MSTN*; the contents of UDCA, α-MCA, β-MCA, HCA, GUDCA, GHDCA, GDCA, and 6-ketoLCA were significantly positively correlated with the expression of *mTOR*. The levels of UDCA, α-MCA, β-MCA, HCA, CA, GHCA, GHDCA, GDCA, and 6-ketoLCA were negatively correlated with the expression of *MYH4*; the concentrations of DCA and DHLCA were significantly negatively correlated with the expression of *MYH2*, *MYH1* *PPARGC1A*, and *SIRT1*; the levels of CDCA, α-MCA, GCDCA, isoHDCA, HDCA, GLCA, GUDCA, and GHDCA were significantly negatively correlated with the expression of *MYOG*; the concentration of DCA was significantly negatively correlated with the expression of *IGF1,AKT*, and *mTOR* (*P* < 0.05, Figure S3A)*.*

The association between fecal BAs and the expression of genes related to lipid metabolism in backfat tissue showed that the abundances of UDCA, CDCA, α-MCA, β-MCA, HCA, CA, GCDCA, THDCA, and 6-ketoLCA were significantly negatively correlated with the mRNA levels of *LPL*, *SREBF1*, and *PPARG,* which was positively correlated with the levels of DCA and TCA-3S; The concentrations of β-MCA, HCA, isoHDCA, and 6-ketoLCA were significantly negatively correlated with the mRNA abundance of *ACACA*; the levels of β-MCA, β-DCA,GHDCA, GDCA, THDC, and TDCA were significantly negatively correlated with the mRNA expression of *CPT1B* (*P* < 0.05, Figure S3B).

***Supplementary tables***

**Table S1** Composition and nutrient levels of the diet

| Items | Diet composition |
| --- | --- |
| Ingredients | |
| Corn | 75.55 |
| Soybean meal (43% CP) | 21.75 |
| Soybean oil | 0.55 |
| Limestone | 1.00 |
| CaHPO_4_ | 0.80 |
| Premix^1^ | 0.35 |
| Total | 100.00 |
| Nutrient levels^2^ | |
| Gross energy /(MJ/kg) | 16.13 |
| Dry matter | 86.81 |
| Crude protein | 14.84 |
| Ether extract | 3.02 |
| Crude ash | 4.16 |
| Total dietary fiber | 12.90 |
| Soluble dietary fiber | 1.62 |
| Insoluble dietary fiber | 11.28 |
| Water binding capacity/(g/g) | 2.25 |

^1^ The premix provided the following per kg of diets0mg: VA 5512 IU, VD_3_ 2200 IU, VE 30 IU, VK_3_ 2.2 mg, VB_1_ 1.5 mg, VB_24_ 3.0 mg, VB_6_ 3.0 mg, VB_12_ 27.6μg, pantothenic acid 14.0mg, nicotinic acid 30.0 mg, choline 400.0 mg, folic acid 0.7 mg, biotin 44.0μg, Mn 40.0 mg, Fe 75.0 mg, Zn 75.0 mg, Cu 20.0 mg, I 0.3 mg, Se 0.

^2^ Nutrient levels were all measured values.

**Table S2** Sequences of primers used for quantitative RT-PCR detection

| Genes symbol | Accession no. | Nucleotide sequence of primers (5′–3′) | Product length |
| --- | --- | --- | --- |
| *ACTB* | XM_021086047.1 | F: TCTGGCACCACACCTTCT | 114 |
|  |  | R: TGATCTGGGTCATCTTCTCAC |  |
| *MYH7* | NM_213855.2 | F: AAGACCCGCTCAACGAGACAGTGG | 121 |
|  |  | R: GCCTTGCCTTTGCCCTTCTCAACA |  |
| *MYH2* | NM_214136.1 | F: TCATCAGTGCCAACCCGCTG | 120 |
|  |  | R: AAGCCAGTTTTCCTGTAGTGCCAAA |  |
| *MYH4* | NM_001123141.1 | F: TTGAGGAGTTAAAGAGGCAGCTAGAAGAGG | 166 |
|  |  | R: TCGCTGTTGGCCTTGGACATTGC |  |
| *MYH1* | NM_001104951.2 | F: TGGAGGCCAGGGTACGTGAA | 134 |
|  |  | R: CTTGCGGTCTTCCTCAGTTTGGT |  |
| *PPARGC1A* | XM_021100444.1 | F: AGACCTGACACAACACGGACAGA | 147 |
|  |  | R: TTCAAGAGCAGCAAAAGCATCACAGG |  |
| *SIRT1* | NM_001145750.2 | F: GAGAAGGAAACAATGGGCCG | 155 |
|  |  | R: ACCAAACAGAAGGTTATCTCGGT |  |
| *MYOD* | NM_002478.5 | F: TCCGACGGCATGATGGATTA | 161 |
|  |  | R: GAGATGCTCTCCACGATGCT |  |
| *MYOG* | NM_002479.6 | F: TCAGCTCCCTCAACCAGGAG | 163 |
|  |  | R: GCTGTGAGCAGATGATCCCC |  |
| *MSTN* | NM_005259.3 | F: GCCTGGAAACAGCTCCTAAC | 158 |
|  |  | R: CGTTTCCGTCGTAGCGTGA |  |
| *IGF1* | XM_054371953.1 | F: TCTTCTACTTGGCCCTGTGC | 141 |
|  |  | R: ACCCTGTGGGCTTGTTGAA |  |
| *AKT* | XM_054375558.1 | F: GCTCTGAGGATGCCAAGGAG | 191 |
|  |  | R: CTCCATGCTGTCGTCTTGGT |  |
| *mTOR* | XM_054335746.1 | F: AGGCGTCTCGCTTGTACTTC | 132 |
|  |  | R: CTCGACCGTATGCCTGGTTA |  |
| *ACACA* | XM_054315912.1 | F: TGTCCACTCAAGCATACCTCCCA | 136 |
|  |  | R: GCTACCATGCCAATCTCATTTCCTCC |  |
| *FASN* | XM_054315477.1 | F: GCCGAGTACAGCGTCAACAACC | 173 |
|  |  | R: TGGTCCTTCTTCATCAGCGGGAT |  |
| *CPT1B* | NM_0002478.1 | F: AGTCATGGTGGGCGACTAACTATGTG | 168 |
|  |  | R: ATCATGGCGTGGACAGCGTTC |  |
| *LPL* | NM_000237.3 | F: AACGTCATTGTGGTGGACTGGCT | 165 |
|  |  | R: TCCAAGGCTGTATCCCAGGAGGTG |  |
| *CD36* | XM_021102279.1 | F: CTGTGGACTCATTGCTGGTGCTG | 179 |
|  |  | R: AAAACTGTCTGTAAACTTCCGTGCCT |  |
| *PNPLA2* | NM_001098605.1 | F: CCTGCCTCTCTACGAACTCAAGAGC | 132 |
|  |  | R: AGGCTGAACTGGATGCTGGTGT |  |
| *SREBP1* | NM_214157.1 | F: CGCAAGACGGCGGATTTA | 218 |
|  |  | R: GCGACGGTGCCTCTGGTAGT |  |
| *PPARG* | XM_005669788.3 | F: TCCAGCATTTCCACTCCACAC | 127 |
|  |  | R: GGGACACAGGCTCCACTTTG |  |

**Table S3** The dominant phyla (average abundance > 0.5%) in M2 and M4 pigs

| Treatment | Phylum | Average abundance |
| --- | --- | --- |
| M2 | *Firmicutes* | 66.15% |
|  | *Bacteroidota* | 13.43% |
|  | *Proteobacteria* | 2.26% |
|  | *Spirochaetota* | 11.03% |
|  | *Desulfobacterota* | 0.91% |
| M4 | *Firmicutes* | 82.32% |
|  | *Bacteroidota* | 7.78% |
|  | *Proteobacteria* | 3.25% |
|  | *Spirochaetota* | 0.62% |
|  | *Desulfobacterota* | 0.75% |

M2, pigs were given two meals per day; M4, pigs were given four meals per day.

**Table S4** The dominant genera (average abundance > 1.0%) in M2 and M4 pigs

| Treatment | Genus | Average abundance |
| --- | --- | --- |
| M2 | *Treponema* | 11.02% |
|  | *Streptococcus* | 6.77% |
|  | *Muribaculaceae* | 5.75% |
|  | *Terrisporobacter* | 5.48% |
|  | *Eubacterium_coprostanoligenes_group* | 4.94% |
|  | *Clostridium_sensu_stricto_1* | 4.92% |
|  | *Lactobacillus* | 4.36% |
|  | *Romboutsia* | 3.02% |
|  | *Prevotellaceae_UCG-003* | 2.87% |
|  | *Christensenellaceae_R-7_group* | 2.67% |
|  | *p-251-o5* | 2.26% |
|  | *Alloprevotella* | 1.45% |
|  | *Family_XIII_AD3011_group* | 1.31% |
|  | *Turicibacter* | 1.21% |
| M4 | *Streptococcus* | 9.12% |
|  | *Clostridium_sensu_stricto_1* | 8.90% |
|  | *Terrisporobacter* | 8.03% |
|  | *Lactobacillus* | 5.76% |
|  | *Romboutsia* | 5.72% |
|  | *Eubacterium_coprostanoligenes_group* | 5.07% |
|  | *Bacillus* | 4.12% |
|  | *Muribaculaceae* | 2.82% |
|  | *Turicibacter* | 2.51% |
|  | *Christensenellaceae_R-7_group* | 1.79% |
|  | *Pedobacter* | 1.46% |
|  | *Escherichia-Shigella* | 1.28% |
|  | *Family_XIII_AD3011_group* | 1.21% |
|  | *Clostridia_UCG-014* | 1.14% |
|  | *Lachnospiraceae_NK4A136_group* | 1.02% |

M2, pigs were given two meals per day; M4, pigs were given four meals per day.***Supplementary figures***


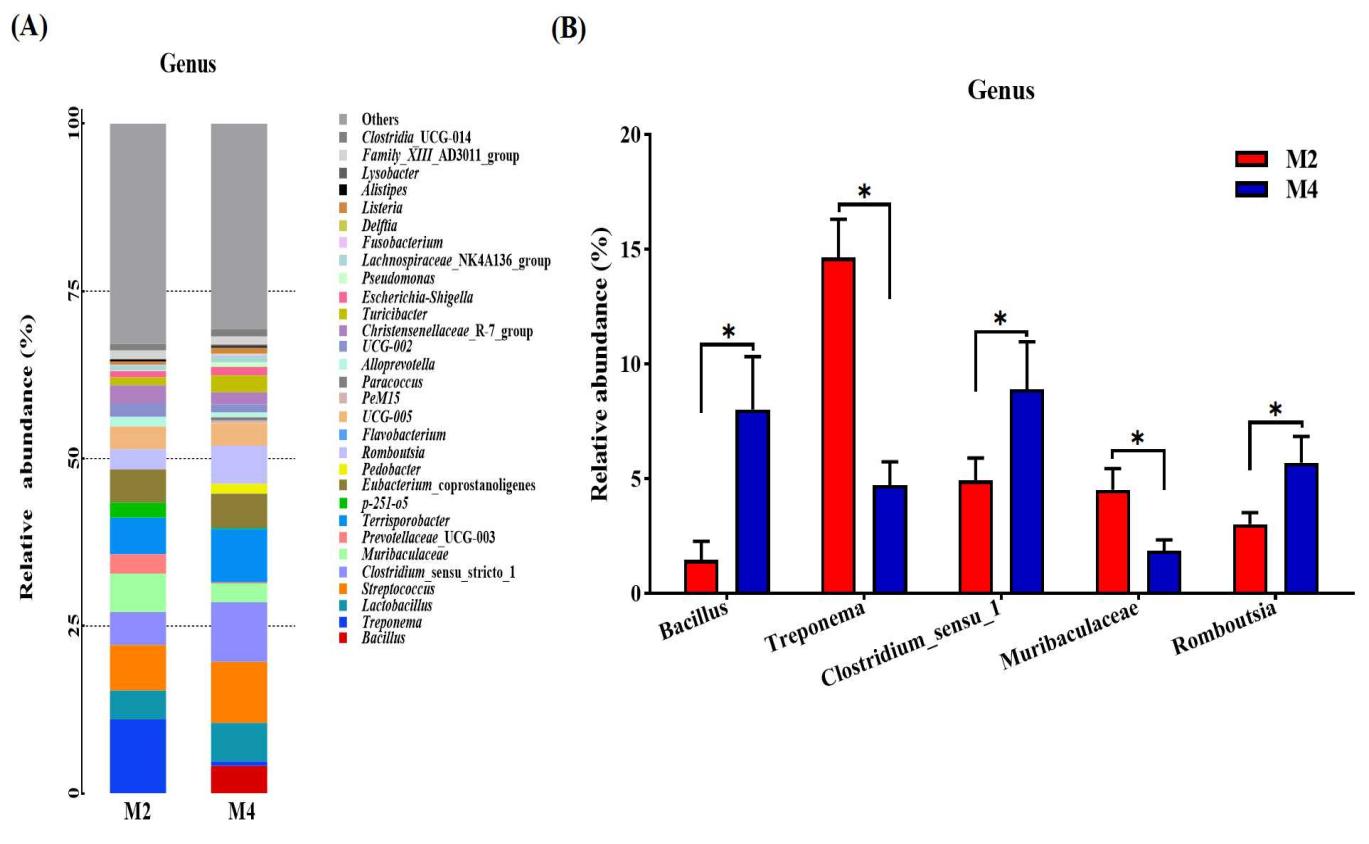


**Figure. S1** Effect of feeding frequency on cecal microbiota composition at the genus level of Sichuan-Tibetan black pigs. (A) The distribution of top 30 genera in both groups; (B) The relative abundance of differentiated genera between groups. M2, pigs were fed two meals per day; M4, pigs were fed four meals per day. **P* < 0.05, n = 8 for each group.


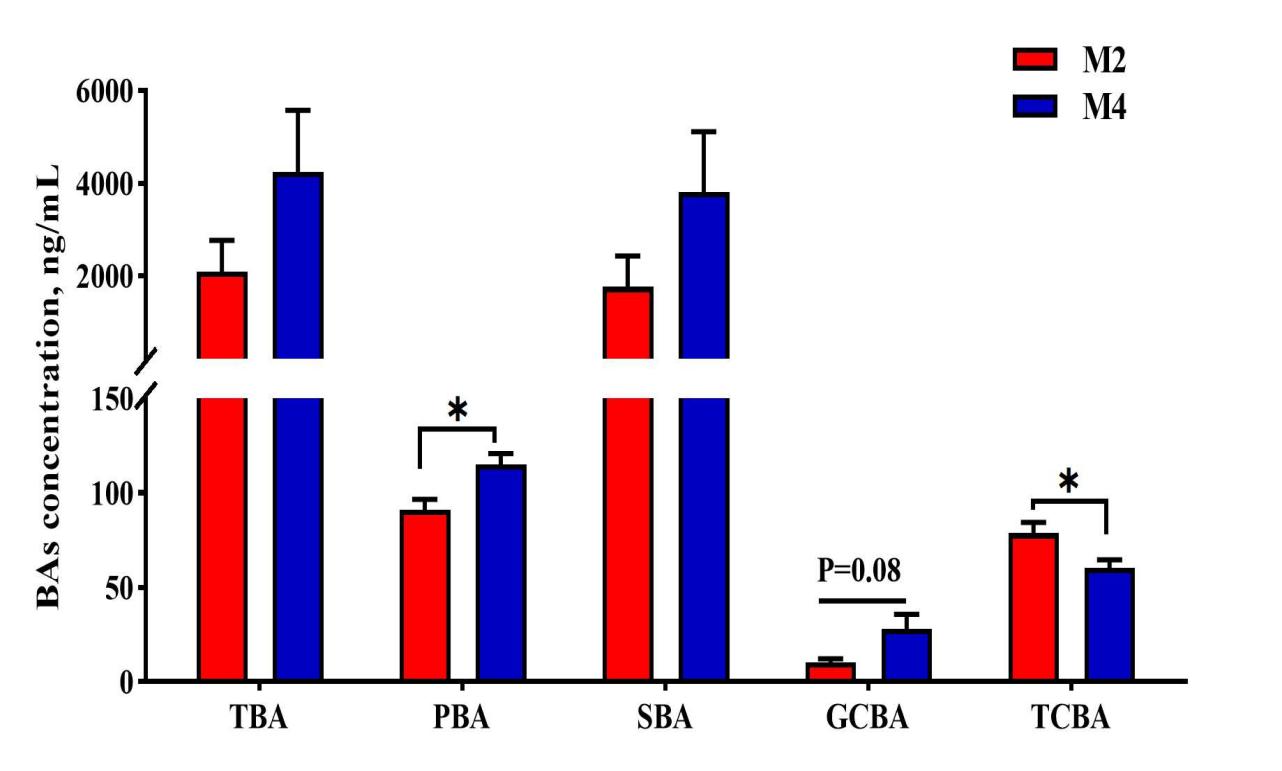


**Figure. S2** Effect of feeding frequency on each type of BA concentration in cecal contents of Sichuan-Tibetan black pigs. TBA, total bile acids; PBA, primary bile acids; SBA, secondary bile acids; GCBA, glycine-conjugated bile acids; TCBA; taurine-conjugated bile acids. M2, pigs were given two meals per day; M4, pigs were given four meals per day. **P* < 0.05, n = 8 for each group.


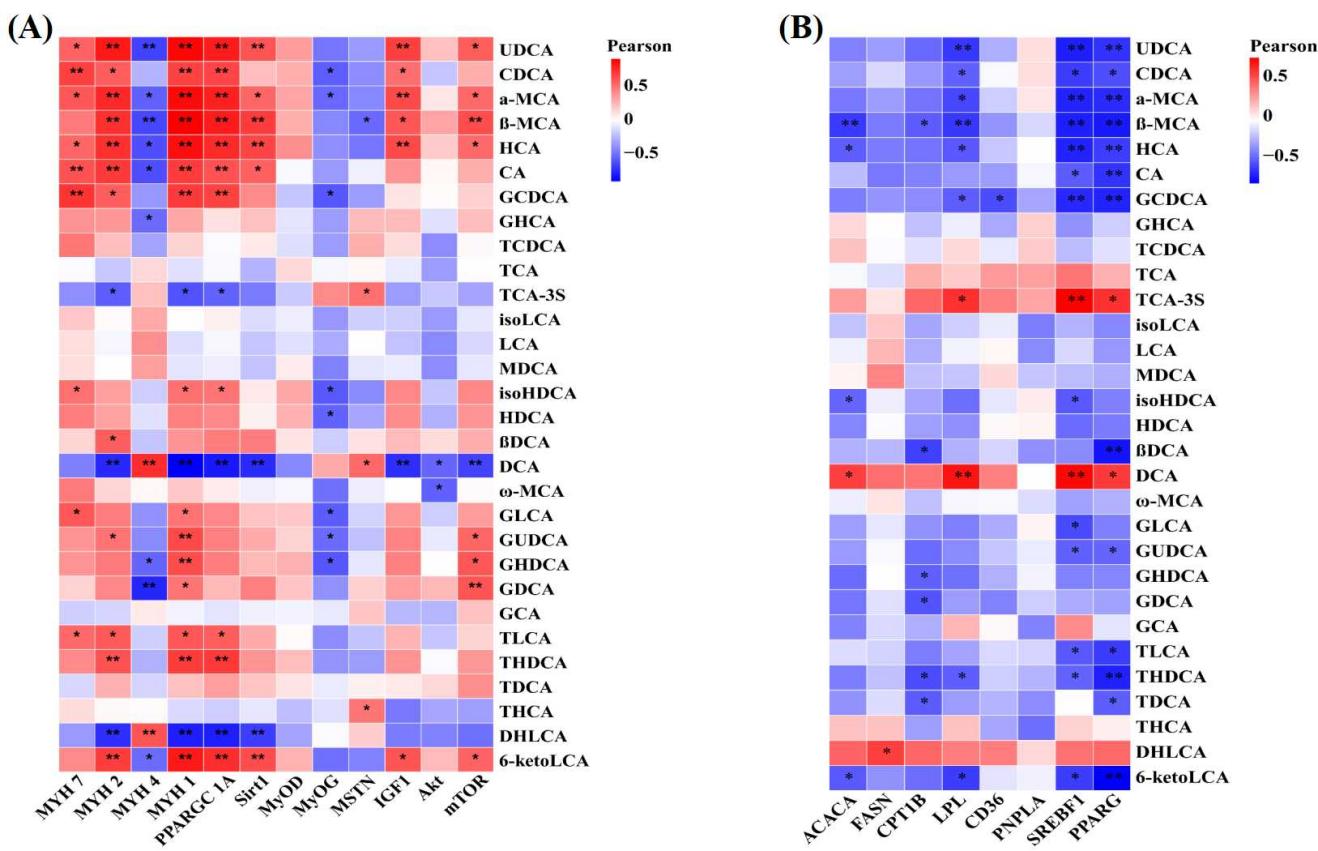


**Figure. S3** Heatmap of the pearson correlation between the abundances of top 30 BAs and the expression of genes related to myofiber transformation and myogenesis (A) and related to lipid metabolism (B). CDCA, chenodeoxycholic acid; α-MCA, α-Muricholic acid; β-MCA, β-muricholic acid; HCA, hyocholic acidCA, Cholic acid; GCDCA, glycochenodeoxycholic acid; GHCA, glycohyocholic acid; TCDCA, taurochenodeoxycholic acid; TCA, taurocholic acid; TCA, taurocholic Acid-3-Sulfate; UDCA, ursodeoxycholic acid; isoLCA, isolithocholic acid; LCA, lithocholic acid; MDCA, murideoxycholic acid; isoHDCA, isohyodeoxycholic acid; HDCA, hyodeoxycholic acid; βDCA, epideoxycholic acid; DCA, deoxycholic acid; ω-MCA, ω-muricholic acid; GHDCA, glycohyodeoxycholic acid; THDCA, taurohyodeoxycholic acid; GLCA, glycolithocholic acid; GUDCA, glycoursodeoxycholic acid; GDCA, glycodeoxycholic acid; GCA, glycocholic acid; TLCA, taurolithocholic acid; TDCA, taurodeoxycholic acid; THCA, taurohyocholic acid; DHLCA, dehydrolithocholic acid; 6-ketoLCA, 6-ketolithocholic acid. *MYH7*, *MYH2, MYH4* and *MYH1,* myosin heavy chain isoforms, *MYH*, myosin heavy chain; PPARGC1A, peroxisome proliferator-activated receptor gamma coactivator 1-alpha; SIRT1, silent information regulator 1; *MYOD*, myogenic differentiation 1; *MYOG*, myogenin; *MSTN*, myostatin; *IGF1*, insulin-like growth factor 1; *AKT*, protein kinase B; *mTOR*, mammalian target of rapamycin; *ACACA*, acetyl-CoA carboxylase alpha; FASN, fatty acid synthase; *CPT1B*, carnitine palmitoyl transferase 1B; *CD36*, CD36 molecule; *LPL*, lipoprotein lipase; *PNPLA2*, patatin-like phospholipase domain containing 2; *SREBF1*, sterol regulatory element binding transcription factor 1; *PPARG*, peroxisome proliferator activated receptor gamma. **P <* 0.05, ***P* *<* 0.01.
